# Impact of long-term feeding a high level of Spirulina combined with enzymes on growth performance, carcass traits and meat quality in broiler chickens

**DOI:** 10.3389/fvets.2024.1451516

**Published:** 2024-08-27

**Authors:** Maria P. Spínola, Mónica M. Costa, Beatriz Tavares, José M. Pestana, João C. Tavares, Cátia F. Martins, Cristina M. Alfaia, Verena Maciel, Daniela F. P. Carvalho, Miguel P. Mourato, Madalena M. Lordelo, José A. M. Prates

**Affiliations:** ^1^CIISA–Centro de Investigação Interdisciplinar em Sanidade Animal, Faculdade de Medicina Veterinária, Universidade de Lisboa, Lisbon, Portugal; ^2^Laboratório Associado para Ciência Animal e Veterinária (AL4AnimalS), Lisbon, Portugal; ^3^Instituto Superior de Agronomia, Universidade de Lisboa, Lisbon, Portugal; ^4^LEAF–Linking Landscape, Environment, Agriculture and Food, Instituto Superior de Agronomia, Universidade de Lisboa, Associated Laboratory TERRA, Lisbon, Portugal

**Keywords:** Spirulina, peptidase, broiler chicken, growth performance, meat quality

## Abstract

This study evaluates the effect of prolonged feeding with a high inclusion level of Spirulina, combined with peptidases, on broiler chicken’s growth performance, digesta viscosity, carcass attributes and meat quality. The experiment involved 120 male broilers divided into 40 battery brooders, each housing 3 birds. Post 7-day acclimatisation with a corn and soybean-based diet, the birds were provided with one of four diets: a corn and soybean meal-based diet (CON), a mix incorporating 15% Spirulina (SP), a Spirulina-rich mix supplemented with 0.025% of commercial VemoZyme^®^ P (SPV), or a Spirulina-rich mix supplemented with 0.10% of porcine pancreatin (SPP). The CON group had higher body weight and weight gain (*p* < 0.001) and a lower feed conversion ratio (*p* < 0.001) from day 7–21, compared to the Spirulina-fed groups. Spirulina-fed chickens significantly increased ileum viscosity (*p* < 0.05). Spirulina also elevated the weight (*p* < 0.05) of the duodenum and the length (*p* < 0.001) of the entire gastrointestinal tract compared to CON. Breast and thigh muscles from Spirulina-fed broilers displayed higher values of yellowness (b*) (*p* < 0.001), pigments (*p* < 0.05), and n-3 PUFA (*p* < 0.01), while n-6/n-3 ratio (*p* < 0.001) and α-tocopherol (*p* < 0.001) decreased relative to the CON. In conclusion, the introduction of a high level of Spirulina into broiler diets for an extended duration, has the potential to diminish birds’ growth performance, possibly due to increased digesta viscosity. However, it does enhance the nutritional quality of the meat.

## 1 Introduction

The escalating demand for alternative protein sources as substitutes for conventional ingredients, like soybean (1), has stimulated interest in microalgae as a promising candidate. Notably, poultry has significant potential for integrating microalgae into their diets, indicating its potential for broader application in commercial animal feed (2). In particular, Arthrospira platensis, more commonly known as Spirulina, has gained recognition as an advantageous component of poultry nutrition (3). Spirulina’s high protein content, fluctuating between 50 and 70% (4), positions it as a viable alternative or complement to traditional protein sources (5–7).

Spirulina, an edible microalga belonging to the blue-green algae group (*Cyanophyceae* or cyanobacteria), is recognized for its distinct filamentous multicellular structure and resilience in harsh environments (2, 8, 9). Its cell wall, akin to that of Gram-negative bacteria (10), is predominantly comprised of peptidoglycan and lipopolysaccharides, forming a robust multi-layered barrier (11). Aside from its high protein content and the presence of all essential amino acids (12), Spirulina serves as a bountiful reservoir of carbohydrates, a variety of vitamins including pro-vitamin A, vitamin C and vitamin E, as well as a host of minerals such as iron, calcium, chromium, copper, magnesium, manganese, phosphorus, potassium, sodium and zinc (13, 14). Further enriching its nutritional profile, Spirulina also provides essential fatty acids and an array of pigments including chlorophyll a, phycocyanin, carotenes and xanthophylls (4, 15–19).

Despite the nutrient-rich profile of Spirulina, the indigestible nature of its cell wall and the resilience of its protein-pigment complexes, known as phycocyanins, which are tied to microalgal thylakoid membranes, pose challenges to its protein’s bioaccessibility and digestibility (20). To mitigate these issues, recent *in vitro* studies (21, 22) have investigated the potential of peptidases (EC 3.4), with or without prior treatments, in the hydrolysis of Spirulina proteins. Notably, Niccolai et al. (22) observed a remarkable increase of up to 81% in crude protein digestibility of Spirulina when applying a combination of pepsin and pancreatin. Furthermore, Costa et al. (21) confirmed the beneficial effect of pancreatin, especially when preceded by an extrusion pre-treatment, on the hydrolysis of protein potentially associated with phycocyanins. Peptidases catalyse the degradation of peptide bonds, are involved in the degradation of off-function proteins in cells and play an important role in cell homeostasis (23). However, proteins freed from Spirulina biomass could undergo a gelation process, potentially elevating the viscosity of the digesta and thereby entrapping other nutrients, which limits their absorption in monogastric animals (24, 25). This effect was demonstrated by Pestana et al. (25), who noticed a surge in digesta viscosity resulting in hindered growth performance in broilers fed a high level of Spirulina (15% of feed) supplemented with lysozyme (26). A similar observation was made when piglets were fed 10% of the microalga, where Spirulina proteins showed resistance to the activity of endogenous peptidases, consequently reducing protein digestibility (27). The application of 15% of Spirulina in broilers has been already studied, with no negative effect on production performance or meat quality (28).

Although the beneficial role of exogenous enzymes, such as carbohydrate-active enzymes (CAZymes), in enhancing the nutritional value of poultry diets is well-recognized (26, 29, 30), there remains a significant gap in our understanding of the *in vivo* impact of peptidases on augmenting the bioaccessibility of Spirulina proteins for monogastric animals. These enzymes are used extensively for their broad-spectrum activity against various substrates like barley, a key component of broiler diets (31, 32). CAZymes facilitate improved access to endogenous enzymes to their target substrates, by enhancing feed conversion and digestibility, mainly in young animals with immature digestive tracts (29). Similar to CAZymes, supplementing peptidases in diets that contain a high proportion of Spirulina could potentially boost broiler digestibility. One of the mixtures contains peptidase and α-amylase (VemoZyme^®^ P) and the other encompasses pancreatic peptidase, lipase and amylase (porcine pancreatin). Despite limited information available on the function and applicability of VemoZyme^®^ P in poultry nutrition, related enzymes from the same manufacturer, namely VemoZyme^®^ F (a phytase) and VemoZyme^®^ Plus (a complex enzyme preparation including carbohydrases), have been successfully utilized in laying hens (33) and broilers diets (34). Pancreatin, as a diverse enzyme mixture, is anticipated to aid broilers with an immature digestive tract in handling dietary changes, such as a transition from a corn and soybean meal-based diet to a Spirulina-enriched diet. Notably, previous studies employed pancreatin mixtures in broiler diets, demonstrating positive outcomes for fat digestibility (35). However, to our knowledge, the effects of this mixture on the degradation of algal proteins have not been explored yet.

Furthermore, while certain studies have scrutinized the influence of dietary Spirulina incorporation on broiler performance and meat quality (e.g., Pestana et al. (25), Abdelfatah et al. (36), Costa et al. (37), and Hassan et al. (38)), there exists a paucity of research probing into the combination of long-duration feeding periods with high inclusion levels of Spirulina.

Hence, the purpose of this study was to investigate the impact of incorporating 15% Spirulina in broiler diets, either alone or combined with commercial VemoZyme^®^ P or porcine pancreatin extract, on broiler growth performance, gut viscosity, carcass traits and meat quality, spanning from day 7–35.

## 2 Materials and methods

### 2.1 Animal welfare statement

The experimental procedures conducted in this study were performed in compliance with ethical guidelines and regulations. The protocols followed were approved by the Ethics Commission of CIISA/FMV (Centre for Interdisciplinary Research in Animal Health, Faculty of Veterinary Medicine) and the Animal Care Committee of the National Veterinary Authority (Direção Geral de Alimentação e Veterinária, Lisboa, Portugal). Additionally, the study adhered to the principles and specific guidelines outlined in the European Union legislation (2010/63/EU Directive) concerning the use of animals in scientific research. The Animal Welfare Committee of the Higher Institute of Agronomy at the University of Lisbon (ORBEA/ISA) also approved the experimental procedures with animals, and the study was assigned a protocol code number 0421/000/000/2022.

### 2.2 Animals, management and diets

A total of 120 one-day-old male Ross 308 broiler chicks were housed in 40 wired-floor cages for 35 days, following the procedures described in Alfaia et al. (39), Pestana et al. (25), and Costa et al. (40). The birds were reared in a controlled environment room under standard brooding practices, with appropriate lighting conditions. At day 0, room temperature was maintained at 31°C, following 30°C on day 1, and from day 3 to 27, it decreased 1°C every 3 days to 20°C, after it remained constant until the end of the study. The temperature and ventilation in the room were continuously monitored throughout the study period from day 1–35. Also, the temperature at the cage level was monitored from day 1–35. Each cage measured 66 × 66 cm, with 2 drinking nipples and one feeder.

During the initial 7 days, the birds were provided with *ad libitum* access to a corn and soybean-based diet. From day 7–35, the birds were assigned to one of four dietary treatments: 1) a control diet based on corn and soybean meal (CON); 2) a diet containing 15% Spirulina powder (Allmicroalgae, Pataias, Portugal) (SP); 3) a diet containing 15% Spirulina powder supplemented with 0.025% of the commercial enzyme mix VemoZyme^®^ P (VEMO, Sofia, Bulgaria) (SPV); and 4) a diet containing 15% Spirulina powder supplemented with 0.10% of porcine pancreatin extract (Merck, Darmstadt, Germany) (SPP). The VemoZyme^®^ P enzyme had a proteolytic activity of 15,000 tyrosine units and an amylase activity of 400 units, as stated by the manufacturer. The porcine pancreatin extract contained 350 FIP-U/g protease, 6,000 FIP-U/g lipase and 7,500 FIP-U/g amylase. All diets were formulated to meet the nutrient requirements defined by the NRC (41), and the compositions and nutrient content analysis of the starter, and grower diets plus microalga powder are provided in Tables 1, 2, respectively.

**Table 1:** Ingredient composition and nutrient content analysis of broiler experimental starter diets (day 7–14) (%, as-fed basis).

	CON	SP	SPV	SPP
Ingredients, %				
Corn	50.9	58.3	58.3	58.3
Soybean meal	41.8	23.2	23.2	23.2
Sunflower oil	3.40	0.0	0.0	0.0
Sodium chloride	0.400	0.400	0.400	0.400
Calcium carbonate	1.15	1.47	1.47	1.47
Dicalcium phosphate	1.80	1.10	1.10	1.10
DL-Methionine	0.150	0.050	0.050	0.050
L-Lysine	0.0	0.060	0.060	0.060
Vitamin-mineral premix^1^	0.400	0.400	0.400	0.400
Spirulina powder	0.0	15.0	15.0	15.0
VemoZyme^®^ P	0.0	0.0	0.025	0.0
Porcine Pancreatin	0.0	0.0	0.0	0.100
Nutrient content^2^				
Gross energy, MJ/kg DM	19.1	18.5	18.3	18.2
Crude Protein, % DM	25.5	25.5	26.0	25.7
Crude fat, % DM	6.65	3.87	4.10	3.98
Ash, % DM	7.14	8.93	8.76	8.54

CON, Corn and soybean meal-based diet, in the control group; SP, 15% Spirulina; SPV, 15% Spirulina + 0.025% VemoZyme^®^ P; SPP, 15% Spirulina + 0.10% Porcine pancreatin.

1 Premix provided the following per kilogram of diet: pantothenic acid 10 mg, vitamin D_3_ 2,400 IU, cyanocobalamin 0.02 mg, folic acid 1 mg, vitamin K_3_ 2 mg, nicotinic acid 25 mg; vitamin B_6_ 2 mg, vitamin A 10000 UI, vitamin B_1_ 2 mg, vitamin E 30 mg, vitamin B_2_ 4 mg, Cu 8 mg, Fe 50 mg, I 0.7 mg, Mn 60 mg, Se 0.18 mg, Zn 40 mg.

2 Nutrient content analysed.

**Table 2:** Ingredient composition and nutrient content analysis of broiler experimental grower diets and microalga powder (day 14–35) (%, as-fed basis).

	Spirulina	CTR	SP	SPV	SPP
Ingredients, %					
Corn	–	55.6	62.2	62.2	62.2
Soybean meal	–	36.9	18.6	18.6	18.6
Sunflower oil	–	4.00	1.00	1.00	1.00
Sodium chloride	–	0.300	0.400	0.400	0.400
Calcium carbonate	–	1.08	1.39	1.39	1.39
Dicalcium phosphate	–	1.60	0.900	0.900	0.900
DL-Methionine	–	0.120	0.030	0.030	0.030
L-Lysine	–	0.0	0.050	0.050	0.050
Vitamin-mineral premix^1^	–	0.400	0.400	0.400	0.400
Spirulina powder	–	0.0	15.0	15.0	15.0
VemoZyme^®^ P	–	0.0	0.0	0.025	0.0
Porcine Pancreatin	–	0.0	0.0	0.0	0.100
Nutrient content^2^					
Gross energy, MJ/kg DM	18.7	19.1	18.5	18.5	18.4
Crude Protein, % DM	52.5	23.6	23.5	23.1	23.3
Crude fat, % DM	6.56	7.88	5.19	5.30	5.14
Ash, % DM	21.6	6.41	8.21	8.27	8.19
Calcium, % DM	1.20	1.42	1.87	2.21	2.01
Available phosphorus, % DM	0.001	0.710	0.593	0.700	0.660
Sodium, % DM	4.51	0.299	0.860	1.03	0.94
Fatty acid profile, % total fatty acids					
14:0	0.432	0.072	0.078	0.119	0.113
16:0	39.7	9.90	16.1	16.2	15.9
16:1c9	4.95	0.133	0.796	0.797	0.776
17:0	0.410	0.065	0.105	0.122	0.123
17:1c9	1.88	0.035	0.215	0.222	0.221
18:0	1.75	3.05	3.19	2.56	2.58
18:1c9	6.34	31.8	27.1	26.7	27.4
18:2n-6	17.8	51.1	45.4	46.3	45.9
18:3n-3	15.8	1.27	3.36	3.43	3.46
20:0	0.189	0.379	0.415	0.426	0.431
20:1c11	0.9106	0.160	0.204	0.206	0.215
22:0	0.247	0.580	0.383	0.391	0.393
Diterpene profile, μg/g					
α-Tocopherol	29.5	58.1	57.3	51.1	47.5
α-Tocotrienol	–	4.37	4.36	3.77	4.29
*β*-Tocopherol		0.512	0.360	0.367	0.342
γ-Tocopherol + *β*-Tocotrienol	–	7.74	6.96	7.46	6.81
γ-Tocotrienol	–	10.9	10.1	10.6	10.2
δ-Tocopherol	–	0.904	0.752	0.687	0.751
Pigments, μg/g DM					
*β*-Carotene	294	1.34	115	121	107
Chlorophyll a^3^	1,600	13.3	504	517	538
Chlorophyll b^4^	1,404	26.8	74.0	67.0	70.9
Total chlorophylls^5^	3,004	40.0	578	584	608
Total carotenoids^6^	447	2.17	152	154	161
Total chlorophylls and carotenoids^7^	3,450	42.2	730	738	769
Minerals (mg/kg DM)					
Macrominerals					
Calcium (Ca)	12,010	14,202	18,735	22,143	20,110
Magnesium (Mg)	8,894	1955	1,526	1912	1869
Potassium (K)	3,230	11,961	10,587	11,501	11,161
Sulphur (S)	67.5	2,929	3,098	3,537	3,261
Total	69,405	41,135	48,476	56,403	52,458
Microminerals					
Copper (Cu)	946	13.2	15.3	16.7	18.4
Iron (Fe)	129,528	156	241	317	284
Manganese (Mn)	67.5	71.0	79.0	96.0	92.3
Zinc (Zn)	99.0	89.3	76.8	93.6	89.3
Total	131,697	329	412	523	484
Total macro and microminerals	201,101	41,464	48,888	56,926	52,942

CON, Corn and soybean meal-based diet, in the control group; SP, 15% Spirulina; SPV, 15% Spirulina +0.025% VemoZyme^®^ P; SPP, 15% Spirulina +0.10% Porcine pancreatin.

1 Premix provided the following per kilogram of diet: pantothenic acid 10 mg, vitamin D¬32,400 IU, cyanocobalamin 0.02 mg, folic acid 1 mg, vitamin K3 2 mg, nicotinic acid 25 mg; vitamin B6 2 mg, vitamin A 10000 UI, vitamin B1 2 mg, vitamin E 30 mg, vitamin B2 4 mg, Cu 8 mg, Fe 50 mg, I 0.7 mg, Mn 60 mg, Se 0.18 mg, Zn 40 mg.

2 Nutrient content analysed.

3 Ca: Chlorophyll a = 11.24 × A662 nm−2.04 × A645 nm.

4 Cb: Chlorophyll b = 20.13 × A645 nm−4.19 × A662 nm.

5 Ca + b: Total chlorophylls = 7.05 × A662 nm + 18.09 × A645 nm.

6 Cx + c: Total carotenoids = (1,000 × A470 nm−1.90 × Ca−63.14 × Cb) / 214.

7 Ccc: Total chlorophylls and carotenoids = (Ca + b) + (Cx + c).

The experimental design consisted of 10 replicate cages, with 3 birds per cage. Weekly measurements of broiler and feeder weights were recorded, and feed was provided daily to calculate body weight gain (difference between body weight of two consecutive weeks, divided by 7), average daily feed intake (weekly ingestion by each cage, divided by 7) and feed conversion ratio (ratio between weekly ingestion divided by 3 and weekly body weight gain). Diet samples were analysed for dry matter (DM) by drying a sample at 103°C until a constant weight was achieved. The nitrogen (N) content of the diets was determined using the Kjeldahl method according to AOAC method 954.01 (42), and crude protein content was calculated as 6.25 times the nitrogen content. Ash content was determined using AOAC method 942.05 (42). Crude fat was determined by extracting feed samples with petroleum ether using an automatic Soxhlet extractor (Gerhardt Analytical Systems, C. Gerhardt GmbH & Co. KG, Königswinter, Germany), following a prior hydrolysis with hydrochloric acid. The gross energy of the feed was determined using adiabatic bomb calorimetry (Parr 1,261, Parr Instrument Company, Moline, IL, United States).

On day 35, one bird from each cage, with the middle weight, was subjected to electrical stunning and manual exsanguination. Blood samples were collected in Sarstedt tubes (Numbrecht, Germany) and centrifuged to obtain serum. The gastrointestinal (GI) organs (crop, gizzard, duodenum, jejunum, ileum and cecum) were removed and emptied, and the weight of the crop, gizzard, liver, pancreas, duodenum, jejunum, ileum and cecum was recorded. The length of the duodenum, jejunum, ileum, and cecum was also measured. Duodenum in the proximal part of the intestinal tract, with a “U” shape around the pancreas. The jejunum is the distal part of the duodenum. Anterior to the junction of the cecum, is the ileum. The cecum is a paired tubular structure distal along the ileum from the ileo-caecal-colic junction (43). The viscosity of the contents of the small intestine was determined following the method described by Pestana et al. (25). Briefly, samples were collected from the duodenum plus jejunum and ileum, centrifuged for 10 min at 9.000 rpm, and the viscosity of the supernatant was measured using a viscometer (Model LVDVCP-II, Brookfield Engineering Laboratories, Middleboro, MA, United States) at a room temperature maintained at 24°C. Samples of breast and thigh muscles (deboned and skinless) were chopped and kept in aluminium foil, bagged in vacuum bags and stored at −20°C until analyses.

### 2.3 Determination of carcass traits

The pH and colour analysis of the meat were conducted following the methods described in Pestana et al. (25) and Alfaia et al. (39). Briefly, the right breast (pectoralis major) and thigh (biceps femoris) muscles were deboned and skinned. Triplicate measurements were taken from three different spots on each muscle. The pH values were determined using a glass penetration pH electrode (HI9025, Hanna Instruments, Woonsocket, RI, United States), while colour measurements [lightness (L*), redness (a*), and yellowness (b*)] were obtained using a Minolta CR-300 Chromameter (Minolta Camera Co. Ltd., Osaka, Japan) based on the CIELAB colour space. The data were recorded after a 24-h cooling post-mortem period at 4°C, followed by 1 h of air exposure.

### 2.4 Sensorial panel traits of breast meat

The sensory analysis was performed following the procedures outlined in Pestana et al. (25). In brief, the right skinless breast muscles were individually cooked in a water bath at 85°C using plastic bags until the internal temperature reached 78°C. For the sensory evaluation, the cooked muscle samples were trimmed of external connective tissue and cut into approximately 1 cm^3^ cubes. These cubes were then kept at 60°C on pre-identified heated plaques. A panel of 10 trained assessors, selected and trained according to Cross (44) at the Faculty of Veterinary Medicine (University of Lisbon, Portugal), evaluated the samples. The sensory panel sessions consisted of five sessions, with eight random samples per session. The attributes assessed were tenderness, juiciness, off-flavours and overall acceptability. A structured 8-point scale was used for the sensory evaluation, with 1 representing extremely tough, dry, weak, and negative attributes, and 8 representing extremely tender, juicy, strong, and positive attributes for tenderness, juiciness, flavour and overall acceptability, respectively.

### 2.5 Determination of meat lipid oxidative stability

Approximately 1.5 g of minced meat from the left breast of each bird was divided into four portions and placed in plastic bags. These portions were then exposed to air and stored in a freezer at 4°C for 0 and 8 days. The concentration of thiobarbituric acid reactive substances (TBARS), an indicator of lipid oxidation, was measured on day 0 and day 8. The spectrophotometric method described by Mercier et al. (45) was followed to analyse the ability of malondialdehyde, a product of lipid oxidation, to form a pink-coloured chromogen that absorbs 532 nm light. The measurements were taken using a UV/visible spectrophotometer (Genesys 150, ThermoScientific, Madison, United States). For TBARS quantification, a standard calibration curve was prepared using 1,1,3,3-tetraethoxypropane (Fluka, Neu Ulm, Germany) as a precursor of malondialdehyde. The results are expressed as milligrams of malondialdehyde per kilogram of meat.

### 2.6 Determination of total cholesterol, diterpenes, pigments and minerals in meat and experimental diets

The determination of total cholesterol, *β*-carotene and tocopherols in both fresh meat (750 mg) and feed (100 mg) followed the protocol described by Prates et al. (46), with specific details provided in Pestana et al. (25), Alfaia et al. (39), Costa et al. (40). The measurements were performed in duplicate, and the concentrations were calculated based on the external standard technique using a standard curve of peak area versus concentration.

For the analysis of chlorophyll a, chlorophyll b and total carotenoids, the method outlined by Teimouri et al. (47) was employed, with slight modifications described in Pestana et al. (25), Alfaia et al. (39), and Costa et al. (40). All procedures related to pigment extraction and analysis were conducted under dim light conditions to minimize the photodegradation of pigments. The contents of pigments were determined using equations described by Hynstova et al. (16).

To determine the mineral profile, including calcium (Ca), potassium (K), magnesium (Mg), sodium (Na), phosphorus (P), sulphur (S), copper (Cu), iron (Fe), manganese (Mn) and zinc (Zn), the procedures outlined in Ribeiro et al. (48) and Costa et al. (40) were followed. Inductively coupled plasma optical emission spectrometry (ICP-OES) with an iCAP 7,200 duo instrument (Thermo Scientific, Waltham, MA, United States) was utilized for the analysis. Calibration curves using multi-element standards (PlasmaQual S22, SPC Science, Baie-D’Urfé, QC, Canada) were employed to quantify the different elements.

### 2.7 Determination of dry matter and total lipids in meat

The dry matter content of breast and thigh meats was determined following the method described by Rosenkranz (49). Freeze-dried samples were obtained by using a freeze-drier (Labogene, CoolSafe, Lillerod, Denmark) at −60°C and 2.0 hPa. The freeze-drying process was carried out until a constant weight was achieved. After freeze-drying, the samples were stored in desiccators at room temperature until further analysis.

To determine the total lipids in the meat samples, as well as in the feed, the freeze-dried breast and thigh muscles were subjected to lipid extraction according to the procedure outlined by Folch et al. (50). The extraction was performed using a mixture of dichloromethane and methanol in a ratio of 2:1 (v/v). The extracted lipids were then evaporated to dryness, and the fatty residue obtained was weighed gravimetrically. The measurements were performed in duplicate.

### 2.8 Determination of fatty acid composition in meat and experimental diets

The fatty acid composition of breast and thigh muscles was determined by converting the fatty acid residue into fatty acid methyl esters (FAME). This conversion was carried out using a combined basic and acidic transesterification procedure. Firstly, the fatty acids were transesterified using NaOH in anhydrous methanol (0.5 M) at 50°C for 30 min. Subsequently, HCl/methanol (1:1 v/v) was used for a second transesterification step at 50°C for 10 min (51). The same procedure was applied for the analysis of FAME in feed samples, except that direct transesterification was performed using HCl/methanol (1:1 v/v) at 70°C for 2 h.

The analysis of FAME was conducted following the method described by Pestana et al. (25). Gas chromatography (GC System, 7890A, Agilent Technologies, California, United States) comprising a Supelcowax^®^ 10 capillary column (30 m × 0.20 mm internal diameter, 0.20 μm film thickness; Supelco, Bellefonte, PA, United States) and flame ionization detector, was employed for the analysis, using specific parameters. The injector temperature and detector temperature were set at 250°C and 280°C, respectively. Helium gas was used as the carrier gas at a flow rate of 1.0 mL/min, with a split ratio of 1:20. The gas chromatograph oven temperature was programmed as follows: an initial temperature of 50°C was maintained for 4 min, followed by a ramp of 13°C/min to 175°C (maintained for 20 min), and then a ramp of 4°C/min to 275°C (maintained for 44 min).

Identification of FAME was based on a comparison of retention times with a standard (FAME mix 37 components, Supelco Inc. Bellefonte, PA, United States) confirmed by gas chromatography coupled to mass spectrometry using a GC–MS QP2010-Plus (Shimadzu, Kyoto, Japan). Quantification of FAME was achieved using heneicosanoic acid (21:0) methyl ester as the internal standard. The resulting fatty acid composition was expressed as g/100 g of total fatty acids.

### 2.9 Statistical analysis

All the data were analysed using the Generalized Linear Mixed (GLM) model of the SAS program (SAS Institute Inc., Cary, NC, United States) for most variables, except for the TBARS values, the repeated measures in time (PROCMIXED) procedure of SAS were employed. The cage served as the experimental unit for body weight, body weight gain, average daily feed intake and feed conversion ratio, while the individual bird was considered as the experimental unit for gastrointestinal organs weight and length, and content viscosity and meat quality measurements. To determine statistical differences among dietary treatments, a significant multiple comparisons test was conducted using the PDIFF option, which was adjusted with the Tukey–Kramer method. A *p*-value less than 0.05 was considered statistically significant to indicate meaningful differences.

## 3 Results

### 3.1 Growth performance and gastrointestinal tract parameters

Table 3 compiles the data on birds’ growth performance and gastrointestinal tract parameters in response to the different dietary conditions. Birds subjected to Spirulina diets manifested diminished body weights (BW) on day 35, in contrast to those sustained on the control diet (CON) (*p* < 0.001). The pattern persisted throughout the treatment span (days 14, 21, 28 and 35), wherein birds fed with Spirulina (SP, SPV and SPP) consistently showed lesser gains in body weight (BWG) and lower average daily feed intake (ADFI) relative to the control group (CON) (*p* < 0.001).

**Table 3:** Growth performance and gut content viscosity of broilers.

	CON	SP	SPV	SPP	SEM	*p*-value
Body weight, g						
Day 7	126	129	132	125	4.23	0.712
Day 14	339 ^a^	268^b^	261^b^	255^b^	8.42	<0.001
Day 21	663^a^	447^b^	449 ^b^	447^b^	18.0	<0.001
Day 28	1,041^a^	685^b^	698 ^b^	696^b^	28.9	<0.001
Day 35	1,501^a^	998^b^	1,009^b^	1,035^b^	44.0	<0.001
Body weight gain, g						
Day 7–14	30.8^a^	19.8^b^	18.5^b^	18.6^b^	0.775	<0.001
Day 14–21	45.8^a^	25.6^b^	26.8 ^b^	27.4^b^	1.60	<0.001
Day 21–28	54.0^a^	34.0^b^	35.6^b^	35.6^b^	2.02	<0.001
Day 28–35	65.9^a^	44.8^b^	44.5^b^	48.5^b^	2.73	<0.001
Day 7–35	50.9^a^	32.2^b^	32.5^b^	33.7^b^	1.55	<0.001
Average daily feed intake, g/pen						
Day 7–14	114^a^	93.7^b^	90.2^b^	89.7^b^	2.70	<0.001
Day 14–21	197^a^	132^b^	136^b^	140^b^	6.08	<0.001
Day 21–28	257^a^	173^b^	181^b^	184^b^	9.57	<0.001
Day 28–35	321^a^	234^b^	246^b^	256^b^	13.5	<0.001
Day 7–35	222^a^	158^b^	163^b^	167^b^	7.29	<0.001
Feed conversion ratio						
Day 7–14	1.30^b^	1.67^a^	1.75^a^	1.67^a^	0.0433	<0.001
Day 14–21	1.55^b^	1.84^a^	1.78^a^	1.8 ^a^	0.0503	<0.001
Day 21–28	1.71	2.02	1.79	2.33	0.300	0.470
Day 28–35	1.76	1.90	2.17	1.89	0.127	0.159
Day 7–35	1.61^b^	1.78^a^	1.81^a^	1.83^a^	0.0364	<0.001
Content viscosity, cP						
Duodenum + jejunum	4.26	5.45	6.07	5.76	0.526	0.123
Ileum	6.23^b^	11.8^a^	12.8^a^	12.8^a^	0.903	<0.001

SEM, standard error of the mean.

^a,b^ Different superscripts within a row indicate a significant difference (*p* < 0.05).

CON, Corn and soybean meal-based diet, in the control group; SP, 15% Spirulina; SPV, 15% Spirulina +0.025% VemoZyme^®^ P; SPP, 15% Spirulina +0.10% Porcine pancreatin.

Similarly, the feed conversion ratio (FCR) displayed higher values for the Spirulina-fed groups (SP, SPV and SPP), as compared to the control group (CON) (*p* < 0.001). However, this trend was interrupted between days 21–28 and 28–35, wherein no significant FCR differences were noted. In addition, birds fed Spirulina, irrespective of enzyme supplementation, presented with increased viscosity in the ileum contents relative to their counterparts in the control diet (CON) (*p* < 0.001).

Despite these trends, most gastrointestinal (GI) tract organ weights remained largely unaffected by the dietary treatment (*p* > 0.05), as outlined in Table 4. Exceptions included the relative weights of the pancreas (*p* < 0.001) and duodenum (*p* < 0.05), which increased across all Spirulina-fed groups (SP, SPV and SPP). Moreover, the relative lengths of the duodenum, jejunum, ileum and caecum were greater in all Spirulina-fed groups (SP, SPV and SPP) (*p* < 0.001).

**Table 4:** Relative weight and length of the gastrointestinal tract of broilers.

	CTR	SP	SPV	SPP	SEM	*p*-value
Relative weight of GI^1^ tract, g/kg BW						
Crop	3.51	5.00	4.92	5.37	0.573	0.127
Gizzard	17.2	18.6	18.5	16.3	1.26	0.510
Liver	23.8	21.5	21.7	22.2	1.07	0.439
Pancreas	2.58^b^	3.13^a^	3.36^a^	3.08^a^	0.119	<0.001
Duodenum	5.87^b^	6.55^ab^	7.29^a^	7.46^a^	0.346	0.009
Jejunum	11.4	10.2	11.8	10.6	0.644	0.289
Ileum	9.78	9.71	11.0	10.9	0.407	0.108
Caecum^2^	4.46	5.52	4.73	5.45	0.349	0.094
Relative length of GI tract, cm/kg BW						
Duodenum	20.2^b^	28.2^a^	29.1^a^	28.1^a^	1.53	<0.001
Jejunum	45.3^b^	63.5^a^	66.4^a^	64.7^a^	3.33	<0.001
Ileum	53.5^b^	72.6^a^	72.2^a^	73.8^a^	3.52	<0.001
Caecum	12.2^b^	15.6^a^	15.7^a^	16.5^a^	0.874	0.007

SEM, standard error of the mean.

^a,b^ Different superscripts within a row indicate a significant difference (*p* < 0.05).

CON, Corn and soybean meal-based diet, in the control group; SP, 15% Spirulina; SPV: 15% Spirulina +0.025% VemoZyme^®^ P; SPP, 15% Spirulina +0.10% Porcine pancreatin.

1 GI, Gastrointestinal.

2 Caecum, weight of 2 caecum.

### 3.2 Carcass traits, meat quality and sensory evaluation

Table 5 presents a detailed analysis of the impact of dietary treatments on the carcass traits and meat quality of broiler birds. The study found no significant variance in the lightness (L*) and redness (a*) of the breast and thigh meats across the different dietary treatments (*p* > 0.05). However, significant differences were observed in terms of pH24h (*p* < 0.05) and yellowness (b*) (*p* < 0.001), both of which were markedly higher in birds fed Spirulina as compared to those subjected to CON.

**Table 5:** Carcass traits and meat quality of broilers’ breast and thigh meats fed with Spirulina.

	CON	SP	SPV	SPP	SEM	*p*-value	CON	SP	SPV	SPP	SEM	*p*-value
	Breast meat						Thigh meat					
pH24h	5.81^a^	5.57^b^	5.56^b^	5.55^b^	0.034	<0.001	6.06^a^	5.93^ab^	5.96^ab^	5.88^b^	0.046	0.051
Lightness (L*)	59.81	57.71	58.99	58.15	0.983	0.453	54.45	54.90	56.20	55.10	0.757	0.419
Redness (a*)	4.40	4.99	5.03	4.86	0.360	0.592	7.13	8.15	8.68	8.92	0.542	0.110
Yellowness (b*)	9.20^b^	20.93^a^	22.87^a^	21.36^a^	0.730	<0.001	10.02^b^	23.59^a^	25.86^a^	25.72^a^	0.878	<0.001

SEM, standard error of the mean.

^a,b^ Different superscripts within a row indicate a significant difference (*p* < 0.05).

CON, Corn and soybean meal-based diet, in the control group; SP, 15% Spirulina; SPV, 15% Spirulina +0.025% VemoZyme^®^ P; SPP, 15% Spirulina +0.10% Porcine pancreatin.

As presented in Table 6, sensory evaluations, conducted by a trained panel for the breast meat, highlighted several significant differences between the diet groups. Birds that were fed Spirulina diets, irrespective of whether these were supplemented with enzymes, exhibited lower scores for meat tenderness, juiciness, and overall acceptability compared to those in the control group (CON) (*p* < 0.001). Nonetheless, the inclusion of both enzymes (SPV and SPP) appeared to counteract the decrease in meat flavour attributed to the Spirulina diets (*p* < 0.05). The addition of pancreatin seemed to particularly aid in reversing the decline in meat tenderness and overall acceptability. Of note, dietary treatments did not result in any significant changes in off-flavours (*p* > 0.05).

**Table 6:** Sensorial panel traits of broiler breast meat.

	CON	SP	SPV	SPP	SEM	*p*-value
Tenderness	6.04^a^	5.00^b^	5.31^b^	5.63^ab^	0.186	<0.001
Juiciness	5.45^a^	4.56^b^	4.44^b^	4.61^b^	0.157	<0.001
Flavour	6.01^a^	5.48^b^	5.59^ab^	5.69^ab^	0.121	0.013
Off-flavour	0.096	0.196	0.151	0.201	0.0706	0.684
Overall	5.85^a^	4.92^b^	5.06^b^	5.31^ab^	0.153	<0.001

SEM, standard error of the mean.

^a,b^ Different superscripts within a row indicate a significant difference (*p* < 0.05).

CON, Corn and soybean meal-based diet, in the control group; SP, 15% Spirulina; SPV, 15% Spirulina +0.025% VemoZyme^®^ P; SPP, 15% Spirulina +0.10% Porcine pancreatin.

The different attributes evaluated in sensorial traits were quantified on a rating scale from 1 (low score) to 8 (high score), except flavour and off-flavour which were quantified from 0 (absence) to 8 (very intense).

### 3.3 Oxidative stability, diterpenes and total pigments

Table 7 presents the findings on the oxidative stability of broiler breast meat across various dietary treatments over two time periods (day 0 and day 8). Oxidative stability is gauged by the measurement of TBA reactive substances, specifically MDA levels. It is worth noting that no significant interaction was found between dietary treatment and period (*p* > 0.05). On both day 0 and day 8, the MDA levels across all dietary treatments did not demonstrate significant differences (*p* > 0.05). However, a noteworthy exception was observed with the SPV treatment, which showed a marked increase in MDA levels between day 0 and day 8 (*p* < 0.001). This indicates that there might be an increase in the oxidation rate of broiler meat within this specific dietary group over time.

**Table 7:** Oxidative stability of broiler breast meat measured as TBA reactive substances.

Malondialdehyde, mg/kg	CON	SP	SPV	SPP	SEM	*p*-value		
						Diet	Period	Diet*Period
Day 0	0.17^x^	0.18^x^	0.14^x^	0.15^x^	0.021	0.137		
Day 8	0.31^abx^	0.27^bx^	0.56^ay^	0.30^abx^	0.087		<0.001	0.059

SEM, standard error of the mean.

^a,b^ Different superscripts within a row indicate a significant difference (*p* < 0.05).

^x,y^ Different superscripts between rows indicate a significant difference (*p* < 0.05).

CON, Corn and soybean meal-based diet, in the control group; SP, 15% Spirulina; SPV, 15% Spirulina +0.025% VemoZyme^®^ P; SPP, 15% Spirulina +0.10% Porcine pancreatin.

Table 8 depicts the impact of different dietary treatments on the diterpene profile and pigments in the meat of broilers. The data indicate a marked decrease in the levels of diterpenes, specifically α-tocopherol and γ-tocopherol+*β*-tocotrienol, in both breast and thigh meats of broilers fed with Spirulina-based diets when compared to the control group (CON) (*p* < 0.001). Moreover, the minor diterpenes, α-tocotrienol and *β*-tocopherol, were detected in breast meat but were absent in thigh meat. Conversely, the levels of pigments, including total chlorophylls, total carotenoids and their combined sum, were significantly elevated in the broiler breast and thigh meats from the Spirulina-fed groups (SP, SPV and SPP) compared to the control group (CON) (all *p* < 0.001, except *p* = 0.003 in total chlorophylls in breast meat). Among the Spirulina-fed groups, those combined with enzymes (SPV and SPP groups), exhibited higher levels of *β*-carotene in breast meat compared to the control group (CON) (*p* = 0.008), although no significant differences were found in the thigh muscle.

**Table 8:** Diterpene profile and pigments of broiler meats.

	CON	SP	SPV	SPP	SEM	*p*-value	CON	SP	SPV	SPP	SEM	*p*-value
	Breast meat						Thigh meat					
Diterpene profile, μg/g												
α-Tocopherol	10.5^a^	2.20^b^	2.53^b^	2.00^b^	0.426	<0.001	9.63^a^	3.80^b^	4.41^b^	3.61^b^	0.428	<0.001
γ-Tocopherol+*β*-Tocotrienol	0.11^a^	0.05^b^	0.06^b^	0.05^b^	0.010	<0.001	0.114^a^	0.060^bc^	0.075^b^	0.056^c^	0.005	<0.001
α-Tocotrienol	0.13	0.24	0.16	0.18	0.063	0.663	nd	nd	nd	nd	nd	nd
*β*-Tocopherol	0.026^a^	0.018^b^	0.018^b^	0.018^b^	0.001	<0.001	nd	nd	nd	nd	nd	nd
*β*-Carotene	0.039^b^	0.058^ab^	0.062^a^	0.061^a^	0.005	0.008	nd	0.224	0.175	0.174	0.021	0.156
Pigments, μg/100 g												
Chlorophyll a^1^	22.4^b^	34.6^ab^	40.2^a^	32.2^ab^	4.12	0.033	15.0^b^	36.6^a^	36.5^a^	32.6^a^	1.25	<0.001
Chlorophyll b^2^	27.3^b^	56.4^a^	63.8^a^	53.1^a^	6.17	0.001	25.3^b^	60.8^a^	63.1^a^	56.4^a^	2.37	<0.001
Total chlorophylls^3^	49.8^b^	90.1^a^	104^a^	85.2^ab^	9.80	0.003	40.3^b^	97.4^a^	99.6^a^	89.0^a^	3.54	<0.001
Total carotenoids^4^	29.4^c^	242^b^	288^a^	253^ab^	9.95	<0.001	38.1^b^	230^a^	251^a^	223^a^	12.17	<0.001
Total chlorophyll and carotenoid^5^	79.2^b^	351^a^	392^a^	338^a^	14.86	<0.001	78.4^b^	327^a^	351^a^	312^a^	11.10	<0.001

SEM, standard error of the mean; nd, not detected.

^a,b,c^ Different superscripts within a row indicate a significant difference (*p* < 0.05).

CON, Corn and soybean meal-based diet, in the control group; SP, 15% Spirulina; SPV, 15% Spirulina +0.025% VemoZyme^®^ P; SPP, 15% Spirulina +0.10% Porcine pancreatin.

1 Ca: chlorophyll a = 11.24 × A662 nm−2.04 × A645 nm.

2 Cb: chlorophyll b = 20.13 × A645 nm−4.19 × A662 nm.

3 Ca + b: total chlorophylls = 7.05 × A662 nm + 18.09 × A645 nm.

4 Cx + c: total carotenoids = (1,000 × A470 nm−1.90 × Ca−63.14 × Cb) / 214.

5 Ccc: total chlorophylls and carotenoids = (Ca + b) + (Cx + c).

### 3.4 Meat lipids and fatty acid composition

Table 9 presents the total lipid content, total cholesterol content and fatty acid profile of both breast and thigh meats. The total lipid and total cholesterol contents of breast meat in Spirulina-fed birds were significantly lower (*p* < 0.001) than those fed the control diet (CON). However, the difference was not statistically significant in the thigh meat (*p* = 0.061 for total lipids, and *p* = 0.683 for total cholesterol).

**Table 9:** Total lipid content, cholesterol content and fatty acid profile of broiler meats.

	CON	SP	SPV	SPP	SEM	*p*-value	CON	SP	SPV	SPP	SEM	*p*-value
	Breast meat						Thigh meat					
Total lipids, g/100 g	1.35^a^	0.81^b^	0.95^b^	0.88^b^	0.079	<0.001	2.90	2.32	2.39	2.45	0.162	0.061
Cholesterol, mg/g	0.79^a^	0.61^b^	0.67^b^	0.62^b^	0.025	<0.001	0.76	0.81	0.77	0.82	0.043	0.683
Fatty acid composition, g/100 g fatty acids												
12:0	0.02	0.04	0.03	0.03	0.004	0.067	0.02	0.02	0.02	0.03	0.002	0.076
14:0	0.33	0.37	0.37	0.35	0.017	0.300	0.42^b^	0.49^ab^	0.50^a^	0.46^ab^	0.020	0.036
14:1c9	0.05^b^	0.06^a^	0.06^a^	0.07^a^	0.017	0.006	0.06^b^	0.10^a^	0.10^a^	0.09^a^	0.006	0.001
15:0	0.08^b^	0.10^ab^	0.10^ab^	0.12^a^	0.006	0.034	0.08^b^	0.11^a^	0.10^ab^	0.12^a^	0.007	0.018
16:0	16.6^b^	20.7^a^	21.0^a^	20.8^a^	0.286	<0.001	18.4^b^	22.1^a^	22.1^a^	21.1^a^	0.683	0.002
16:1c7	0.44	0.55	0.52	0.59	0.036	0.058	0.54^b^	0.74^a^	0.72^a^	0.71^a^	0.037	0.002
16:1c9	1.43^b^	2.18^a^	2.32^a^	2.14^a^	0.172	0.003	2.28^b^	3.90^a^	4.05^a^	3.77^a^	0.244	<0.001
17:0	0.16^b^	0.25^a^	0.25^a^	0.25^a^	0.011	<0.001	0.17^b^	0.27^a^	0.29^a^	0.26^a^	0.018	0.000
17:1c9	0.05^b^	0.09^a^	0.09^a^	0.09^a^	0.004	<0.001	0.06^b^	0.11^a^	0.10^a^	0.08^b^	0.007	<0.001
18:0	9.10	9.76	9.35	9.72	0.240	0.183	8.05	8.11	7.84	7.72	0.271	0.722
18:1c9	31.4^a^	26.7^b^	27.5^b^	26.0^b^	0.828	0.000	35.8	34.4	35.1	34.7	0.914	0.714
18:1c11	1.45^b^	2.51^a^	2.53^a^	2.62^a^	0.077	<0.001	1.39^b^	2.02^a^	2.09^a^	1.99^a^	0.074	<0.001
18:2n-6	29.1^a^	21.5^b^	21.7^b^	21.9^b^	0.484	<0.001	27.3^a^	20.8^b^	20.2^b^	21.5^b^	1.37	0.003
18:3n-6	0.09	0.07	0.10	0.07	0.009	0.046	0.07^a^	0.06^b^	0.05^b^	0.06^b^	0.004	0.001
18:3n-3	0.45^b^	0.56^ab^	0.58^a^	0.56^ab^	0.031	0.033	0.46^b^	0.75^a^	0.72^a^	0.75^a^	0.049	0.000
20:0	0.10	0.11	0.11	0.11	0.006	0.359	0.10	0.09	0.09	0.09	0.005	0.636
20:1c11	0.26^b^	0.33^ab^	0.35^ab^	0.38^a^	0.027	0.019	0.27	0.28	0.30	0.29	0.013	0.381
20:2n-6	0.43^b^	0.50^b^	0.50^b^	0.65^a^	0.038	0.002	0.23	0.19	0.18	0.21	0.019	0.357
20:3n-6	0.68^b^	1.28^a^	1.24^a^	1.38^a^	0.063	<0.001	0.38	0.52	0.50	0.51	0.040	0.069
20:4n-6	4.48^b^	6.69^a^	6.05^a^	7.00^a^	0.364	<0.001	1.86	2.36	2.16	2.24	0.260	0.572
20:3n--3	0.02^c^	0.05^b^	0.05^b^	0.06^a^	0.004	<0.001	0.02	0.03	0.02	0.03	0.003	0.133
20:5n-3	0.04^b^	0.14^a^	0.13^a^	0.16^a^	0.012	<0.001	0.02^b^	0.05^a^	0.04^ab^	0.04^a^	0.006	0.005
22:0	0.07^b^	0.10^a^	0.09^ab^	0.10^ab^	0.006	0.029	0.05	0.04	0.03	0.04	0.003	0.058
22:1n-9	0.03	0.04	0.05	0.04	0.005	0.060	0.04	0.06	0.07	0.04	0.014	0.194
22:5n-3	0.22^b^	0.60^a^	0.54^a^	0.63^a^	0.037	<0.001	0.04	0.06	0.05	0.06	0.005	0.208
22:6n-3	0.15^b^	0.46^a^	0.42^a^	0.46^a^	0.029	<0.001	0.09^b^	0.25^a^	0.20^a^	0.20^ab^	0.029	0.003
Others	2.63	4.02	3.73	3.52	0.409	0.111	0.06^b^	0.16^a^	0.16^a^	0.15^a^	0.021	0.004
Partial sums of fatty acids												
SFA	26.5^b^	31.7^a^	31.5^a^	31.6^a^	0.379	<0.001	27.4^b^	31.3^a^	31.0^a^	29.9^ab^	0.831	0.008
MUFA	35.2	32.4	33.5	32.0	0.992	0.130	40.5	41.6	42.5	41.7	1.19	0.682
PUFA	35.7^a^	31.9^b^	31.3^b^	32.9^b^	0.738	0.001	30.5	25.1	24.3	25.7	1.64	0.049
n-6 PUFA	34.8^a^	30.1^b^	29.6^b^	31.0^b^	0.704	<0.001	29.8^a^	23.9^ab^	23.1^b^	24.5^ab^	1.60	0.022
n-3 PUFA	0.88^b^	1.81^a^	1.72^a^	1.87^a^	0.053	<0.001	0.66^b^	1.24^a^	1.15^a^	1.17^a^	0.079	<0.001
Nutritional ratios												
n-6/n-3	39.9^a^	16.8^b^	17.3^b^	16.6^b^	0.611	<0.001	44.4^a^	19.7^b^	20.5^b^	22.1^b^	1.71	<0.001
PUFA/SFA	1.35^a^	1.01^b^	1.00^b^	1.04^b^	0.029	<0.001	1.16^b^	0.81^a^	0.79^a^	0.87^ab^	0.077	0.005

SEM, standard error of the mean; nd, not detected.

^a,b,c^ Different superscripts within a row indicate a significant difference (*p* < 0.05).

CON, Corn and soybean meal-based diet, in the control group; SP, 15% Spirulina; SPV, 15% Spirulina +0.025% VemoZyme^®^ P; SPP, 15% Spirulina +0.10% Porcine pancreatin.

SFA = 12:0 + 14:0 + 15:0 + 16:0 + 17:0 + 18:0 + 20:0 + 22:0.

MUFA = 14:1c9+ 16:1c7 + 16:1c9 + 17:1c9 + 18:1c9 + 18:1c11 + 20:1c11 + 22:1n−9.

PUFA = 18:2n-6 + 18:3n-6 + 18:3n-3 + 20:2n-6 + 20:3n-6 + 20:4n-6 + 20:3n-3 + 20:5n-3 + 22:5n-3 + 22:6n-3.

n-3 PUFA = 18:3n-3 + 20:3n-3 + 20:5n-3 + 22:5n-3 + 22:6n-3.

n-6 PUFA = 18:2n-6 + 18:3n-6 + 20:2n-6 + 20:3n-6 + 20:4n-6.

For the fatty acid profile of breast meat, there was a significant increase (*p* < 0.001, in general) in the levels of several fatty acids (16:0, 17:0, 14:1c9, 16:1c9, 17:1c9, 18:1c11, 20:3n-6, 20:4n-6, 20:3n-3, 20:5n-3, 22:5n-3 and 22:6n-3), saturated fatty acids (SFA), and n-3 polyunsaturated fatty acids (n-3 PUFA) in Spirulina-fed birds. Additionally, Spirulina-fed groups (SP, SPV and SPP) led to decreased content of 18:2n-6 (*p* < 0.001), total PUFA (*p* = 0.001), n-6 PUFA (*p* < 0.001), n-6/n-3 (*p* < 0.001), and PUFA/SFA (*p* < 0.001) ratios. An interesting observation was a tendency for the content of 18:3n-6 to decrease with SP or SPP diets, and an increase with the SPV diet (*p* = 0.046). SPV and SPP diets also significantly increased 18:3n-3 (*p* = 0.033) and 20:2n-6 (*p* = 0.002) contents, respectively.

Similarly, for the thigh meat, Spirulina-fed birds exhibited increased contents of certain fatty acids (16:0, 17:0, 14:1c9, 16:1c9, 17:1c9, 18:1c11, 18:3n-3 and n-3 PUFA) compared to the CON group (majority *p* < 0.001). However, 18:2n-6 (*p* = 0.003), 18:3n-6 (*p* = 0.001) content, and n-6/n-3 ratio (*p* = 0.022) were significantly lower in Spirulina-fed groups (SP, SPV and SPP). There was a potential decrease in total PUFA content (*p* = 0.049) with the inclusion of Spirulina. The thigh meat of birds fed SP and SPV showed higher contents of 22:6n-3 (*p* = 0.003), SFA (*p* = 0.008), and a significantly lower PUFA/SFA ratio (*p* = 0.005) than the control (CON). The 20:5n-3 content was significantly increased by SP and SPP diets (*p* = 0.005), whereas SPV significantly reduced n-6 PUFA content (*p* = 0.022).

### 3.5 Mineral composition

The mineral composition of breast and thigh meats of broilers subjected to the different diets is presented in Table 10. The influence of Spirulina-enriched diets is seen to have differing effects on the mineral composition of breast and thigh meats. For the breast meat, both zinc content and overall micromineral concentration significantly decreased as a result of Spirulina integration (*p* < 0.001). Conversely, the copper content increased in the breast meat from broilers fed the SPP diet in comparison to the control group (CON) (*p* = 0.030). In the case of thigh meat, there was a significant decrease in the levels of phosphorus (*p* = 0.014), total macrominerals (*p* = 0.016), zinc (*p* < 0.001) and total minerals (*p* = 0.014) in groups fed Spirulina (SP, SPV and SPP), whereas the sodium and total micromineral content exhibited an increase (*p* = 0.007 and *p* < 0.001, respectively). A decrease in magnesium and potassium was also observed in the thigh meat of birds that were fed SPV and SPP diets (*p* = 0.010 and *p* = 0.029, respectively).

**Table 10:** Mineral composition of broiler meats fed with Spirulina.

	CON	SP	SPV	SPP	SEM	*p*-value	CON	SP	SPV	SPP	SEM	*p*-value
	Breast meat						Thigh meat					
Macrominerals (mg/100 g fresh weight)												
Calcium (Ca)	17.8	18.3	17.8	18.7	0.51	0.60	18.3	18.7	18.4	18.8	0.37	0.73
Magnesium (Mg)	25.8	26.9	26.2	26.1	0.45	0.35	20.4^a^	19.1^ab^	18.8^b^	18.7^b^	0.38	0.01
Phosphorous (P)	254	261	263	261	4.1	0.35	246^a^	234^b^	234^b^	235^b^	2.9	0.01
Potassium (K)	408	420	422	419	5.0	0.18	392^a^	375^ab^	376^ab^	375^b^	4.6	0.03
Sodium (Na)	101	94.5	92.6	96.1	2.80	0.15	102^b^	109^a^	109^a^	111^a^	1.9	0.01
Sulphur (S)	209	201	201	201	3.0	0.12	217	208	207	206	3.0	0.06
Microminerals (mg/100 g fresh weight)												
Copper (Cu)	0.12^b^	0.16^ab^	0.17^ab^	0.17^a^	0.01	0.03	0.21	0.21	0.23	0.22	0.006	0.09
Iron (Fe)	1.26	1.30	1.30	1.35	0.03	0.30	1.46	1.45	1.38	1.44	0.028	0.18
Manganese (Mn)	0.06	0.06	0.06	0.06	0.00	0.95	0.06	0.06	0.06	0.06	0.003	0.41
Zinc (Zn)	1.15^a^	0.70^b^	0.71^b^	0.70^b^	0.03	<0.001	1.99^a^	1.64^b^	1.59^b^	1.56^b^	0.046	<0.001
Total	2.59^a^	2.21^b^	2.23^b^	2.27^b^	0.04	<0.001	3.71^a^	3.36^b^	3.26^b^	3.29^b^	0.060	<0.001
Total macro and microminerals	1,019	1,025	1,026	1,024	10.0	0.96	999^a^	968^b^	968^b^	967^b^	7.9	0.01

SEM, standard error of the mean; nd, not detected.

^a,b^ Different superscripts within a row indicate a significant difference (*p* < 0.05).

CON, Corn and soybean meal-based diet, in the control group; SP, 15% Spirulina; SPV, 15% Spirulina +0.025% VemoZyme^®^ P; SPP, 15% Spirulina +0.10% Porcine pancreatin.

To put the above results into perspective, for instance, the zinc level in breast meat declined from 1.15 mg/100 g in the CON to 0.698 mg/100 g in the SPP group. Similarly, in the thigh meat, the phosphorus content was reduced from 246 mg/100 g in the CON to 234 mg/100 g in the SP and SPV groups. However, sodium content in the thigh meat increased from 102 mg/100 g in the CON to 111 mg/100 g in the SPP group.

## 4 Discussion

The presence of protein and pigment complexes attached to the thylakoid membrane (20, 52) in Spirulina, makes the hydrolysis of algal proteins difficult. This associated with the recalcitrance of microalga cell walls, compromises nutrients’ bioaccessibility and bioavailability (12). To improve the bioaccessibility of microalga nutrients in broilers, this study incorporated two commercial enzyme mixtures into the diets.

Spirulina is frequently used as a supplement or ingredient in poultry diets, with incorporation levels ranging between 0.25 and 20% (12). Notably, Valente et al. (53) suggested that microalga incorporation below 15% can augment meat quality without compromising performance. Conversely, higher incorporation levels may promote biomass gelation and escalate digesta viscosity, thereby potentially hindering nutrient digestibility and bioaccessibility (25, 54). In our study, broilers fed with Spirulina showed significantly lower BW and BWG, coupled with an increased FCR compared to the control groups. These findings echo the results of Pestana et al. (25) in a trial involving the same level of inclusion but over half the period (days 21–35). The present results can be attributed to the increased digesta viscosity in the digestive tract, particularly in the ileum, as suggested by Pestana et al. (25). This may have been due to an increase in digesta viscosity, compromising nutrient digestibility. However, interestingly, the FCR remained unaffected by the inclusion of the microalga in the diet from day 21–35. Moreover, an increase in the weight of the duodenum and the length of the duodenum, jejunum and ileum, as observed in Spirulina-fed groups (SP, SPV and SPP), could be a result of the increase in digesta viscosity. The enhanced viscosity can significantly influence nutrient digestibility, bioavailability and accessibility, leading to compromised animal growth and performance (55). In addition, it was reported that higher intakes of Spirulina (above 1.5%) may impact metabolic functions and slow growth rates in broilers (56), while smaller percentages can either improve or have no effect on poultry growth (57, 58). Further, high incorporation levels of Spirulina (up to 21%) can lead to protein gelation and increased digesta viscosity (24, 25), which in turn affects protein and amino acid digestibility and stalls animal growth (59). The discrepancies among various published studies may stem from factors like diverse Spirulina growing conditions, variable inclusion levels in diets and nutritional composition (57). Interestingly, in our study, the enzymatic supplementation in the diet did not reverse the decreased growth performance associated with microalga incorporation. However, the addition of pancreatin slightly mitigated the negative impact on body weight gain from day 28–35. The increase in digesta viscosity caused by high levels of Spirulina proteins in the diet may have trapped the enzymes, thereby inhibiting their action on feed nutrients, a phenomenon that warrants further investigation. An investigation by our group with Spirulina and combination of two types of pre-treatments, such as extrusion method or enzymatic (pancreatin and lysozyme) have shown good results in combining pre-treatments in growth performance, with similar FBW and BWG to control (37).

In the poultry industry, meat colour holds significant importance from the consumer’s perspective. The colour preferences vary widely depending on factors like geographical location, dietary preferences and environmental conditions (60). The supplementation of Spirulina, particularly in concentrations ranging from 4.0 to 15%, has been noted to enhance the redness and yellowness of poultry meat while reducing its lightness. The elevated redness may be attributed to the high iron and mineral content, and the increased yellowness could be due to the greater proportion of carotenoids, such as *β*–carotene and zeaxanthin present in the flesh (60–62). Our study further reinforced these findings, with Spirulina incorporation deepening the yellowness of both breast and thigh meats. This aligns with previous studies by Toyomizu et al. (60), Venkataraman et al. (63), and Altmann et al. (64). Notably, the latter demonstrated that diets supplemented with Spirulina led to more pronounced colour pigmentation of the skin, breast, and thigh meat in broilers. In a study by Moujahed et al. (65) it was observed that incorporating 2.5 and 5% Spirulina into the diet enhanced the yellowness of the breast and skin colour in Arbor Acres broilers. However, a lower level of Spirulina supplementation (0.25, 0.5, 0.75 and 1%) in the diet of Ross 308 male broilers did not significantly affect the colour of the breast meat (58). These differences are not only perceptible in instrumental analysis but also noticeable to the naked eye (64). An orange hue in poultry meat may be perceived as advantageous by consumers when Spirulina is incorporated into broilers’ diets. Meat colour is a significant quality indicator in several countries, particularly in Japan (66), the United States, and Mexico (67). Interestingly, when the breast muscles were cooked for the sensory panel, no discernible differences between samples were found. For the sensory panel, the overall acceptance of all diets was positive (scores between 5 and 6), and the absence of any significant difference in off-flavours between treatment groups, positions Spirulina-fed chicken favourably when compared to meats from birds fed on fish oil diets (68) or those rich in docosahexaenoic acid from algal products, which may affect off-flavours in meat.

Substituting corn-based diets with Spirulina necessitates careful analysis. Spirulina is a novel ingredient incorporated in diets, so it is important to do randomized, double-blind and placebo clinical trials to test the microalga’s functionality (13). The latter’s higher PUFA content could contribute to lipid oxidation, thereby negatively impacting meat quality (69, 70). The PUFA content directly influences lipid oxidation (71), rendering thigh meat. This portion contains more lipids than breast meat, more prone to lipid oxidation (72, 73). Despite the greater lipid and PUFA content in thigh meat, we focused our analysis on TBARS in breast meat due to its prominence in consumers’ diets. Levels of TBARS exceeding 0.8 mg/kg can indicate rancidity and adversely affect meat acceptability because of the resultant oxidized flavour, which is not acceptable to consumers (57, 74). In our study, TBARS levels in breast meat ranged from 0.14 to 0.56 mg/kg, significantly below this threshold.

Spirulina is a microalga renowned for its high content of antioxidants such as carotenoids, *β*-carotene, and tocopherols (75), which can protect lipids from oxidation (76). Our research indicates that the high levels of total carotenoids in both types of poultry meat may reflect Spirulina’s antioxidant capabilities and its ability to enhance the oxidative stability of broiler meat. The high carotenoid content in chickens fed Spirulina could explain the increase in meat yellowness, as a greater proportion of carotenoids in the meat can result in heightened yellowness. In the thigh meat of Spirulina-fed groups (SP, SPV, and SPP), the α-tocopherol and γ-tocopherol+*β*-tocotrienol content was higher than in the breast meat, likely due to the thigh’s higher fat content (77). Nevertheless, the decreased value of α-tocopherol in Spirulina-fed groups compared to the control might be attributed to several factors. Firstly, the oxidative stress induced by the higher polyunsaturated fatty acids (PUFAs) in Spirulina could increase the consumption of α-tocopherol as an antioxidant to counteract lipid peroxidation. Secondly, Spirulina’s high phycocyanin content may provide an additional antioxidant effect, thereby reducing the need for α-tocopherol and causing its levels to decrease as it is utilized more rapidly in oxidative processes. Despite the reduction, the α-tocopherol levels in Spirulina-fed groups remain within the typical range found in chickens (77, 78). Of the vitamin E diterpenes, α-tocopherol was found in the highest concentrations, while other homologues were present at lower levels, consistent with typical values in broilers (77, 78). Therefore, our results indicate that incorporating 15% Spirulina into the diet can enhance the antioxidant function of broiler meat through its high carotenoid content and possibly through synergistic effects with its other antioxidant compounds.

Breast and thigh meats are recognized as lean meats due to their low-fat content, which is generally less than 5% (79). In this study, these criteria were met as the total lipid content ranged from 0.82 to 1.35% in the breast and 2.32 to 2.90% in the thigh. The thigh meat exhibited a higher cholesterol content than the breast, surpassing values reported by Pestana et al. (25). However, the introduction of Spirulina led to a decrease in the cholesterol values of breast meat.

The fatty acid profile was found to be affected by Spirulina in both types of meat, corroborating previous findings by Cortinas et al. (80) and Botsoglou et al. (81). The most abundant fatty acids across all diets were SFA, such as palmitic (16:0) and stearic (18:0) acids, monounsaturated fatty acids (MUFA), like palmitoleic (16:1c9) and oleic acid (18:1c9), n-3 PUFA α-linolenic acid (18:3n-3), and n-6 PUFA, such as linoleic acid (18:2n-6) and arachidonic acid (20:4n-6). These fatty acids are also the most prevalent in Spirulina. The incorporation of Spirulina in the diet increased the SFA content in both types of meat, with palmitic (16:0) and stearic (18:0) acids being predominant. SFA are more resistant to oxidation and are more readily deposited in meats (76). The arachidonic acid content was higher in breast meat that included Spirulina, which could be due to the conversion of linoleic acid (82). Furthermore, the linoleic acid content was reduced in the breast and thigh of broilers fed diets containing Spirulina, and arachidonic acid was not detected in the microalga powder or diets. Both cereal-based feed and Spirulina are weak sources of α-linolenic acid, with the latter containing less than 1% (83). However, the SPV for the breast and SP, SPV and SPP for the thigh, led to an increase in α-linolenic acid, thereby improving the n-3 PUFA content. Due to the competition for desaturase enzymes, which are the same for both n-6 and n-3 pathways, the conversion of essential fatty acids, mainly α-linolenic acid, along the entire n-3 pathway is a limited mechanism (84, 85). Thus, it is crucial to ensure an appropriate dietary linoleic acid/α-linolenic acid ratio to obtain an efficient conversion of α-linolenic acid into beneficial eicosapentaenoic acid (20:5n-3) and docosahexaenoic acid (22:6n-3) (86). In this study, an increase in eicosapentaenoic and docosahexaenoic acids was observed in the breast and thigh meat of broilers fed Spirulina. This suggests a possible conversion of α-linolenic acid into these long-chain PUFA (LC-PUFA), as they were not detected in the microalga. Additionally, the decrease in total PUFA in breast meat, and a trend towards a decrease in the thigh, may suggest an increase in the *de novo* synthesis of fatty acids, facilitated by Δ6 and Δ5 desaturases and elongases (87). Aside from the LC-PUFA content, the fatty acid profile from the breast and thigh meats was consistent with the diets, as reported by other studies (77, 88). The addition of VemoZyme^®^ P and porcine pancreatin did not alter these fatty acid profiles.

The changes in meat fatty acid profile, particularly the increase in n-3 PUFA and the decrease in n-6/n-3 ratio, can be attributed to the composition of Spirulina. Spirulina is a rich source of n-3 fatty acids, including alpha-linolenic acid, which can be converted into longer-chain n-3 PUFA such as eicosapentaenoic acid and docosahexaenoic acid in broilers. The dietary inclusion of Spirulina likely enhanced the deposition of these fatty acids in the meat (4, 89). PUFA play essential roles in functions such as the production and metabolism of prostaglandins, thromboxanes and leukotrienes (89, 90). Dietary guidelines recommend a PUFA/SFA ratio in human diets above 0.45 and an n-6/n-3 ratio not exceeding 4.0 (91). In this study, the guideline for the PUFA/SFA ratio was met, but the n-6/n-3 ratio in breast and thigh meats across dietary treatments significantly exceeded the recommended levels.

Minerals play a significant role in various physiological processes, including maintaining normal body fluids, regulating absorption, secretion and excretion, as well as contributing to skeletal structure and soft tissues (92). Although the integration of Spirulina into the diet did not influence the macro-mineral composition in the breast meat, in the thigh meat, there was a noticeable decrease in magnesium, potassium and total content among groups fed Spirulina (SP, SPV and SPP). Magnesium is a critical player in cellular functions, acting as a cofactor in all major metabolic pathways (93, 94). In contrast, sodium content was higher in groups fed Spirulina (SP, SPV and SPP) compared to the control diet (CON). Sodium and potassium play a crucial role in maintaining water balance, regulating muscle and heart activities, as well as hydrogen ion regulation, and gastric and kidney secretions. They are also integral constituents of blood and body fluids (92).

Regarding micro-minerals, zinc and total content were found to be lower in both breast and thigh meats of the Spirulina-fed groups (SP, SPV and SPP). Zinc, a vital micromineral, is important for the growth and development of broilers, as it influences DNA synthesis and the metabolism of lipids and carbohydrates (95–97). The decrease in zinc levels in the treatment groups may be explained by the high phytic acid content in Spirulina, which is known to bind zinc and reduce its bioavailability. Furthermore, the increased gastrointestinal viscosity due to Spirulina could impair the absorption of minerals, including zinc. These factors likely contributed to the reduced zinc levels observed in the Spirulina-fed groups. Nevertheless, this decreased should be considered if it is higher, because can me associated with severe physical and pathological changes, that influences growth, immune response and reproductive failure (97). Therefore, it is essential to consider the balance of minerals in the diets when incorporating Spirulina to ensure the optimal growth and development of broilers.

## 5 Conclusion

This study demonstrates that incorporating a high level of Spirulina (15%) into broiler diets for an extended period (from day 7–35), with or without enzyme supplementation, negatively impacts broiler growth performance, particularly body weight and body weight gain. The FCR remained unaffected by the inclusion of Spirulina from day 21–35. The increased digesta viscosity observed in Spirulina-fed groups appears to hinder nutrient digestibility, which could explain the compromised growth performance.

In addition, Spirulina integration enhances meat quality by reducing cholesterol content in breast meat and improving the antioxidant profile and n-3 PUFA levels in both breast and thigh meats. These changes potentially make the meat more nutritious and desirable for health-conscious consumers. The addition of commercial enzyme mixtures in this study was not effective in mitigating the increased viscosity in the digestive tract, suggesting that a combination of enzymatic and mechanical or physical pre-treatments may be necessary.

Future research should focus on exploring alternative strategies to improve the digestibility of Spirulina in broiler diets. This could include the use of different enzymatic formulations with specific activity against Spirulina proteins or combined mechanical and enzymatic pre-treatments. Additionally, long-term studies should investigate the potential health benefits of consuming meat enriched with n-3 PUFAs and antioxidants from Spirulina-fed broilers, providing a comprehensive understanding of its implications for both poultry production and human health.

## Data Availability

The raw data supporting the conclusions of this article will be made available by the authors, without undue reservation.
